# Regulation of Adaptive Thermogenesis and Browning by Prebiotics and Postbiotics

**DOI:** 10.3389/fphys.2018.01908

**Published:** 2019-01-10

**Authors:** Bàrbara Reynés, Mariona Palou, Ana M. Rodríguez, Andreu Palou

**Affiliations:** ^1^CIBER Fisiopatología de la Obesidad y Nutrición (CIBERobn), Madrid, Spain; ^2^Institut d'Investigació Sanitària Illes Balears (IdISBa), Palma de Mallorca, Spain; ^3^Laboratory of Molecular Biology, Nutrition and Biotechnology, University of the Balearic Islands, Palma de Mallorca, Spain

**Keywords:** beige adipocytes, brite adipocytes, brown adipose tissue, microbiota, obesity, prebiotics, postbiotics, UCP1

## Abstract

Prebiotics are non-digestible food components able to modify host microbiota toward a healthy profile, concomitantly conferring general beneficial health effects. Numerous research works have provided wide evidence regarding the effects of prebiotics on the protection against different detrimental phenotypes related to cancer, immunity, and features of the metabolic syndrome, among others. Nonetheless, one topic less studied so far, but relevant, relates to the connection between prebiotics and energy metabolism regulation (and the prevention or treatment of obesity), especially by means of their impact on adaptive (non-shivering) thermogenesis in brown adipose tissue (BAT) and in the browning of white adipose tissue (WAT). In the present review, a key link between prebiotics and the regulation of adaptive thermogenesis and lipid metabolism (in both BAT and WAT) is proposed, thus connecting prebiotic consumption, microbiota selection (especially gut microbiota), production of microbiota metabolites, and the regulation of energy metabolism in adipose tissue, particularly regarding the effects on browning promotion, or on BAT recruitment. In this sense, various types of prebiotics, from complex carbohydrates to phenolic compounds, have been studied regarding their microbiota-modulating role and their effects on crucial tissues for energy metabolism, including adipose tissue. Other studies have analyzed the effects of the main metabolites produced by selected microbiota on the improvement of metabolism, such as short chain fatty acids and secondary bile acids. Here, we focus on state-of-the-art evidence to demonstrate that different prebiotics can have an impact on energy metabolism and the prevention or treatment of obesity (and its associated disorders) by inducing or regulating adaptive thermogenic capacity in WAT and/or BAT, through modulation of microbiota and their derived metabolites.

## Introduction

Obesity is one of the major health problems throughout the world (Bray et al., 2017). Current obesity treatment strategies, mainly based on food intake control, seem not to be enough to address this multifactorial disorder of pandemic prevalence. In this sense, studies to counteract obesity development based on energy expenditure are recently booming (Marlatt and Ravussin, 2017). In rodents, browning of white adipose tissue (WAT), and especially adaptive thermogenesis of brown adipose tissue (BAT), are relevant contributors to energy expenditure, being critical for energy balance and body weight maintenance. Uncoupling Protein 1 (UCP1) is the main effector of adaptive (non-shivering) thermogenesis in brown adipocytes, by uncoupling oxidative phosphorylation (Palou et al., 1998; Rodriguez and Palou, 2004). Although UCP1 is mainly expressed in BAT, browning is defined as “any significantly increased UCP1 expression at the mRNA level occurring in what are normally considered as WAT depots” (Nedergaard and Cannon, 2014). In the literature, cells expressing UCP1 in WAT can be referred to as beige or brite (brown-in-white), among other denominations (Nedergaard and Cannon, 2014). In contrast to rodents, adult humans do not exhibit well-defined BAT. However, the presence of discrete areas of active BAT—mainly in the cervical, supraclavicular, axillary, and paravertebral regions, activated in response to certain adrenergic stimuli—has been described in adult humans (Nedergaard et al., 2007). It must be noted that, unlike BAT thermogenesis, the role of beige WAT significantly influencing energy metabolism has been questioned, at least in response to chronic cold and adrenergic agonist treatment, even in the absence of functional interscapular BAT (Labbe et al., 2016, 2018). Nevertheless, although the real relevance of WAT browning to treat metabolic disorders in humans is still unclear (Bartelt and Heeren, 2014), all these findings have opened a new window to study novel treatment strategies of obesity based on enhancing energy expenditure capacity by induction of BAT recruitment and browning of WAT. Cold exposure is the main physiological thermogenic activator, although other physiological situations such as overfeeding can also physiologically activate adaptive thermogenesis (diet-induced thermogenesis) (Rothwell and Stock, 1979; Landsberg et al., 1984). In this sense, the role of the sympathetic nervous system (SNS) is crucial; SNS activation by cold exposure, as well as other thermogenic inductors which promote the release of norepinephrine, stimulates the expression and function of UCP1 in BAT and browning in WAT (Landsberg et al., 1984; Palou et al., 1998; Wu et al., 2012).

Although numerous stimuli, bioactive compounds, and physiological situations have been studied regarding the activation of adaptive thermogenesis, here we will focus on a less explored field, concerning the possible role of prebiotics and microbiota in thermogenic capacity modulation. Microbiota has emerged over the last few years as a key factor modulating different aspects of host health (Villanueva-Millan et al., 2015), and these aspects seem to include adaptive thermogenesis regulation. For instance, a study in mice showed that depletion of gut microbiota of genetically or diet induced obese mice increases browning induction in WAT (Suárez-Zamorano et al., 2015). Moreover, the main physiological activator of adaptive thermogenesis commented above, i.e., cold exposure, has been shown to induce adaptations in gut microbiota profile, which have been related to thermogenic and browning activation. Further, microbiota transplantation of cold exposed mice to germ free mice enhances cold tolerance, related to beige/brown fat formation in WAT (both inguinal and perigonadal) and, to a lesser extent, to BAT recruitment (Chevalier et al., 2015), as we will discuss more extensively below.

Therefore, the relationship between the host microbiota and BAT thermogenesis or WAT browning becomes a focus of interest, even more so when taking into account the fact that the microbiota is physiologically and nutritionally mouldable. One effective physiological way to modulate microbiota, especially gut microbiota, is by the use of pro and prebiotics, especially prebiotics, which can potentially affect to large number of gut microbial species. A prebiotic is defined, at present, as “a substrate that is selectively utilized by host microorganisms conferring a health benefit,” and this modern definition is broad enough to expand the concept from classical carbohydrate molecules to non-carbohydrate compounds, as well as to different applications and sites of action beyond the gastrointestinal tract (Gibson et al., 2017). Sometimes the prebiotic concept has been confused, due to partial overlap in the type of molecules involved, with the concept of “dietary fiber.” Nevertheless, there are significant functional, health-related and structural differences. Dietary fiber has been defined as “non-digestable carbohydrates and lignin that are intrinsic and intact in plants” with “functional fiber” defined as “isolated, non-digestible carbohydrates that have beneficial physiological effects in humans” (Slavin, 2013). Therefore, the definition of fiber is vague in terms of functional/physiological effects and does not include other bioactive compounds distinct to carbohydrates and lignin. The prebiotic concept goes far beyond the fiber concept to include non-carbohydrate substances, applications in different body sites, and compounds from other categories apart from food (Gibson et al., 2017). Importantly, the prebiotic definition includes the requirement of microbiota involvement and selective mechanisms mediated by microbiota, also considering that the health beneficial effects must be documented in order to classify a substance as prebiotic (Gibson et al., 2017). Different mechanisms have emerged to explain the relationship of gut microbiota with health, and these include the modulation of metabolic endotoxemia (referring to lipopolysaccharide—LPS—circulating levels, related to inflammation), gut-endocrine barrier, gut hormones, and peripheral tissue metabolism, among others (Cani and Delzenne, 2010; Geurts et al., 2014; Cani and Everard, 2016). Among the mechanisms involved, the metabolic effects of the products released by the microbiota after their processing, i.e., postbiotics, are gaining a lot of interest and are the focus of specific research. Postbiotics can be defined as “those molecules released by bacteria and other microorganisms that when administered in adequate amount confer health benefits to the host” (Maguire and Maguire, 2018). For instance, short chain fatty acids (SCFA) and different bile acid species are postbiotics that have gained an important focus of attention, as will be discussed below.

Taking into consideration all these antecedents, this review explores the potential link between prebiotics and the regulation of thermogenic capacity and browning, through the modulation of microbiota and postbiotics, as a tool to prevent or combat obesity and its related metabolic disorders. We propose that there is an important impact of microbiota modulation by prebiotics on the regulation of adaptive thermogenic capacity; and that the management of host microbiota by prebiotics might have a prominent role in the thermogenic process, with therapeutic potential.

## Microbiota, Eubiosis, and Dysbiosis

The microbiota is a complex ecosystem of microorganisms, including bacteria, viruses, protozoa and fungi, which live in the human body; about 70% of the microbiota is found in the gastrointestinal tract, where 100 trillion microorganisms coexist, which amounts to more than 10 times the total human cells (Pascale et al., 2018). The most important bacteria populations are in the colon, where a certain symbiosis with the host exists, key for health maintenance (Pascale et al., 2018). Most of the gut microbiota is composed of bacteria, with some predominant *phyla* such as *Firmicutes, Bacteroidetes, Actinobacteria, Verrucomicrobia*, and *Proteobacteria*, and *Methanobacteriales* of the phylum *Euryarchaeota* (Diamant et al., 2011; Chakraborti, 2015). Notably, gut microbiota could be used as a microbiological “fingerprint” since, at the taxonomic level, it exhibits important differences between individuals. In this sense, host genotype, environment and diet influence gut microbiota, responsible for these individual differences (Iebba et al., 2016).

A correct symbiosis between the intestinal microbiota and the host is required for the right production of substances and metabolites necessary for host health maintenance (Boulangé et al., 2016). This correct symbiosis, and the qualitative and quantitative equilibrium between the different species present in the gut microbiota is defined as eubiosis (Iebba et al., 2016), also known as normobiosis, and is key for well-being and health (Petschow et al., 2013). On the other hand, dysbiosis is the term that defines an ecosystem where the microbiota species do not live in harmony, which negatively affects host health; it is usually associated to reduced microbial diversity and the predominance of harmful species, creating a disease-prone situation (Petschow et al., 2013; Zhang et al., 2015).

## Microbiota and Obesity, and Its Metabolic Disorders

As suggested above, a healthy gut microbiota profile contributes to improve host metabolism, through dynamic changes in metabolites, nutrients, and vitamins, and to maintain energy homeostasis (Althani et al., 2016). Meanwhile, alterations in the microbiota profile have been related to the development of some diseases, including obesity and the metabolic disorders that accompany it. In this sense, obese people have been described to present significant differences in the composition of gut microbiota compared to lean people and, concretely, bacteria from the phylum *Bacteroidetes* decrease, whereas bacteria from the phylum *Firmicutes*, such as *Bacillaceae* and *Clostridiaceae*, increase in the obese state, especially related to prolonged exposure to a high-fat (HF) diet (Pascale et al., 2018). The detrimental change in microbiota composition may result in an augmented energy production from undigested materials, leading to a dysregulation in energy homeostasis (Pascale et al., 2018). Moreover, it is also related to other numerous metabolic disturbances, associated with and/or contributing to obesity, such as changes in lipogenesis, triglyceride storage, hepatic steatosis, insulin sensitivity, control of food intake, etc., and the promotion of meta-inflammation (or low grade inflammation) (Cani and Delzenne, 2009). Thus, beyond microbiota involvement in energy utilization from diet, the microbiota seems to play a very relevant role in energy homeostasis, metabolism, and obesity development, thus explaining the increasing interest in analyzing the “obesity-microbiota” link in the last few years (Davis, 2016). Interestingly, differences in intestinal microbiota may even precede overweight development, a conclusion reached for the first time by Kalliomäki et al. (2008). They compared children who remained normal weight with children developing overweight/obesity at the age of 7 years, reporting that the genus *Bifidobacterium* was higher in number at an early age (6–12 months) (when no BMI differences were yet evident) in the posterior normal weight group compared to the overweight group (Kalliomäki et al., 2008). In addition, fecal numbers of *S. aureus* were lower in children remaining normal weight than in children developing overweight (Kalliomäki et al., 2008). These findings strongly reinforce the importance of microbiota in body weight control. In the same line, germ free C57BL/6J male mice fed a HF/high-sucrose diet are protected against diet-induced obesity in contrast to mice with normal gut microbiota (Bäckhed et al., 2007). This resistance to overweight in germ free mice was related to an increase, in liver and skeletal muscle, in the activity of AMP activated protein kinase (AMPK) and key downstream targets (related to the oxidation of fatty acids), together with the induction of the transcriptional co-activator peroxisome proliferator activated receptor gamma co-activator 1α (PGC1α) (another key molecular regulator of energy metabolism) (Bäckhed et al., 2007). Therefore, these findings also support the idea of a marked role of microbiota in energy balance, and provide a clue for the key molecular mechanisms involved.

Bearing in mind this connection between gut microbiota and health, it is clear that prebiotic agents, or other types of gut microbiota modulators, might be considered as agents able to prevent or improve gut dysbiosis and obesity-related metabolic disorders. For instance, *Ganoderma lucidum*, a traditional anti-diabetic medical mushroom, reduces dysbiosis in diet-induced obese mice, related to the decrease in the *Firmicutes/Bacteroidetes* ratio and endotoxin (LPS) levels (Chang et al., 2015). These results were associated with decreased body weight, reduced inflammation, and improved insulin sensitivity in these animals (Chang et al., 2015). Interestingly, the beneficial effects of this prebiotic are transmissible to other diet-induced obese mice through feces transplantation. This suggests that the replacement of a microbial population by a new one, associated with the amelioration of dysbiosis, might confer beneficial health effects to the host (Chang et al., 2015). Recent results with transgenic mice with constitutive production of ω3 polyunsaturated fatty acids (PUFA) also point to the same idea. In this model, the microbiota modulation by ω3 PUFA improves the metabolic profile of high fat (HF) diet fed mice which are protected against obesity, also maintaining a normal gut barrier function and with reduced metabolic endotoxemia (Bidu et al., 2018). Microbiota (fecal) transplantation from the transgenic animals to metabolically altered (by an obesogenic diet) wild type animals is able to improve metabolic profile of the latter and reverse weight gain (Bidu et al., 2018); therefore, these results also suggest an important relationship between gut microbiota and protection against obesity and the related altered metabolism.

## Impact of Gut Microbiota on BAT Thermogenesis and WAT Browning

Different aspects could be important to explain the relationship between gut microbiota and protection against obesity and its metabolic disorders, but when considering the main elements involved in overweight development, one outstanding factor is energy expenditure, where adaptive thermogenesis can play a crucial role. Different research works show an association between modulation of gut microbiota and thermogenic capacity in both BAT and WAT (browning). As far as we know, a link between gut microbiota and BAT metabolism was first shown by Mestdagh et al. (2012). Particularly when comparing conventional and germ-free mice, the authors showed that the absence of gut microbiota activates BAT and hepatic lipid catabolism and inhibits lipogenesis (Mestdagh et al., 2012). Following these lines, other authors have revealed relevant effects of microbiota on BAT thermogenesis and WAT browning, through cold microbiota transplantation experiments. Specifically, Chevalier et al. (2015) showed that cold exposure induces several changes in the microbiota composition and that cold microbiota transplantation (from 6°C exposed C57Bl/6 male mice, for 25 days) to germ free mice induces the expression of the gene coding for UCP1 in BAT, together with increased resting energy expenditure. Likewise, they also revealed that this microbiota transplantation induces browning (with the appearance of UCP1 positive adipocytes) in the inguinal and perigonadal WAT depots. Further supporting the browning phenotype, the expression of some brown/brite markers (*Ucp1, Cidea*, and *Ppargc1a*) (the genes coding for UCP1, cell death-inducing DFFA-Like effector A –CIDEA- and PGC1α) was also induced (Chevalier et al., 2015). Therefore, these studies suggest that cold microbiota transplantation induces browning in WAT and, to a lesser extent, BAT recruitment, possibly related to increased energy dissipation (Chevalier et al., 2015). Similarly, Zietak et al. showed a marked change in microbiota composition in mice exposed to 12°C for 4 weeks, characterized by the increase and decrease in bacterial genera associated to leanness and obesity, respectively, accompanied by an increase in *Ucp1* expression in BAT and WAT (Zietak et al., 2016). Moreover, they also demonstrated that fecal transplantation from cold exposed mice to germ free mice protected the latter from HF-associated disturbances after 6 weeks, increasing protein expression of UCP1 in BAT, reducing body adiposity increase, and improving insulin sensitivity, when compared to germ free mice transplanted with microbiota from 29°C exposed mice. In this case, in the germ free cold microbiota transplanted mice, no *Ucp1* gene expression in inguinal WAT was observed compared to the mice recipient from 29°C microbiota, and the brown phenotype in inguinal WAT was considered minor compared to the marked interscapular BAT thermogenic phenotype observed (Zietak et al., 2016). This was unlike the study of Chevalier et al. discussed above, where browning was considered very relevant (Chevalier et al., 2015). Another interesting question raised by the study of Zietak et al. was the possible role of the modulation of bile acid metabolism by gut microbiota (Zietak et al., 2016), thus connecting with postbiotics (bile acids can act as metabolic regulators derived from microbiota, as will be discussed below). Moreover, Zietak et al. (2017) have pointed out that the functional link between cold and gut microbiota leading to BAT increased thermogenesis is not elucidated yet. As we have previously explained, SNS is crucial for the regulation of thermogenic capacity under cold, diet and other thermogenic inductors (Landsberg et al., 1984; Palou et al., 1998; Wu et al., 2012). Nonetheless, thermogenic capacity (by BAT recruitment or WAT browning) induced by pre- or post-biotics has not necessary been linked to SNS stimulation and adrenergic signaling. In some cases (as we will see for some specific examples below), this link has been suggested, at least as part of the possible mechanistic explanation, but in other cases important evidence supports mechanisms acting by alternative pathways independent of adrenergic signaling (e.g., microbiota derived bile acids) (Zietak et al., 2017). Another condition that correlates changes in gut microbiota with WAT browning, and ameliorates insulin resistance, hepatic steatosis and obesity, is intermittent fasting (in mice), also related to the production of specific postbiotics (acetate and lactate in this case) (Li et al., 2017).

In a model of mice resistant to diet-induced obesity due to enhanced energy expenditure and BAT activity, such as is the β-Klotho KO mouse model, the relationship between microbiota and thermogenic capacity is also suggested. In this model, the usual alteration in gut microbiota profile induced by HF diet feeding is reduced, since β-Klotho KO mice fed a HF diet show a Bacteroidetes/*Firmicutes* ratio similar to wild-type mice fed a chow diet (Somm et al., 2017). The results reported in this work suggest that changes in secondary bile acid production are related to host thermogenesis stimulation (Somm et al., 2017), thus pointing again toward the involvement of bile acids metabolized by bacteria in the thermogenic phenotype, as will be discussed further in the section on postbiotics.

Based on the evidence given so far, it is difficult to establish how much of the energy expenditure increase observed in different models of pre and/or postbiotic supplementation can be attributed specifically to increased adaptive thermogenesis. Nevertheless, all in all, the relationship between gut microbiota and thermogenic capacity (both by BAT recruitment or WAT browning) is evident. Hence, the possibility of modulating microbiota composition by nutritional factors, in this case prebiotics, arises as an interesting physiological way of influencing energy metabolism, concretely adaptive thermogenesis, and prevent or even treat overweight/obesity and the associated metabolic disturbances, as is discussed in the next section.

## Impact of Prebiotics on BAT Thermogenesis and WAT Browning

The concept of prebiotics and the importance of their health promoting effects have already been defined. There are multiple natural sources of prebiotics, such as beans/legumes, starchy fruits, cereals, onions, soybean, etc., and generally multiple kinds of vegetables, as well as other food products such as milk and fungi (Geurts et al., 2014; George Kerry et al., 2018). Moreover, many substances could be considered possible prebiotics, such as galactooligosacaccharides, inulin-type fructans, arabionoxylan, and arabinoxylan oligosaccharides, chitin-glucans from fungi or even several phenolic compounds, given that they favor the growth of some intestinal microorganisms and show potential health effects (Geurts et al., 2014; Markowiak and Slizewska, 2018).

There is extensive literature relating the consumption of diets rich in prebiotics to the improvement of food intake control, and the reduction of body fat content and body weight gain, even in overweight and obese subjects (Roberfroid et al., 2010; Requena et al., 2018). Now, with the present knowledge concerning multiple prebiotics and their effects on gut microbiota, we are able to connect prebiotics with the proposed health effects by the modulation of microbiota, postbiotics, and even deciphering the main mechanisms involved, including the induction of an increased thermogenic phenotype. Therefore, some representative prebiotics able to impact thermogenic capacity will be discussed in this section.

### Phenolic Compounds, Energy Metabolism, and Thermogenesis

Phenolic compounds, or polyphenols, are secondary metabolites naturally produced by plants, which can be classified into two main groups: flavonoids and non-flavonoids. Phenolic compounds are generally characterized by the presence of at least one aromatic ring with one or more hydroxyl groups attached (Crozier et al., 2009). Polyphenols or polyphenol-rich fonts have been described to impact the relative abundance of different bacteria within the gut microbiota in various *in vitro* studies and animal models, by decreasing potential pathogens, such as *C. perfringens* and *C. histolyticum*, and certain Gram-negative *Bacteroides* spp., and inducing beneficial Clostridia, Bifidobacteria and Lactobacilli (Dueñas et al., 2015).

The impact of dietary polyphenols on obesity has also been a topic of interest. Over the years, human and animal studies have revealed that resveratrol, a polyphenol with prebiotic properties, exerts anti-obesity effects (Wang et al., 2015). There are several biological processes involved in the fat-lowering effects of resveratrol, even though the molecular mechanisms implicated have not been fully elucidated (Wang et al., 2015). An important number of studies puts the focus on *in vitro* effects (in cell culture models) of resveratrol, but these types of studies are not the focus of our review. Regarding *in vivo* studies, Alberdi et al. described that resveratrol supplementation induces the expression of some genes involved in BAT activation (*cytochrome-C-oxidase subunit-2*—*Mtco2*–, *Ppargc1a, sirtuin 1*—*Sirt1*–, and *mitochondrial transcription factor A*–*Tfam*), together with protein expression of UCP1, in Sprague-Daley rats fed with an obesogenic diet for 6 weeks (Alberdi et al., 2013). Besides, the combination of resveratrol with another polyphenol, quercetin, also induces UCP1 in BAT (Arias et al., 2017). One possible molecular mechanism involved in BAT activation by resveratrol supplementation is based on AMPK activation (Wang et al., 2017). Nevertheless, part of these conclusions come from the combination of *in vivo* and *in vitro* studies and it is difficult to separate the possible direct effects of resveratrol from its putative effects as a prebiotic agent. In the same line, related to the activation of thermogenesis in BAT, dietary supplementation with resveratrol and quercetin also resulted in the stimulation of browning in WAT (Arias et al., 2017). Namely, the authors showed that resveratrol and quercetin supplementation for 6 weeks in Wistar rats fed with an obesogenic diet induced the appearance of brown-like adipocytes in perirenal WAT (Arias et al., 2017). These studies highlight the link between resveratrol, with its fat lowering effects, and browning and thermogenesis activation. Other interesting study with resveratrol supplementation and WAT browning was performed with oral supplementation of newborn mice throughout lactation, which is a critical period for metabolic programming and the shaping of gut microbiota (Pico and Palou, 2013; Milani et al., 2017). In such study, Serrano et al. (2018) supplemented mice with resveratrol (or vehicle) from day 2 to 20 of life and, in the adulthood (day 90) half of the animals were subjected to an HF diet for 10 weeks. The neonatally male treated animals did no show induction of thermogenic markers in BAT (where expression of *Ucp1* was decreased), but they showed resistance to weight gain and a metabolic programming toward browning in subcutaneous WAT (Serrano et al., 2018). Regarding the issue of the possible effects of resveratrol acting as a prebiotic, it must be highlighted that the direct action of resveratrol modulating gut microbiota has been demonstrated. For example, resveratrol supplementation has been associated with the attenuation of the *Firmicutes*/*Bacteroidetes* ratio and the selective growth of potential beneficial genera such as *Lactobacillus* and *Bifidobacterium*, either alone or in combination with quercetin (Larrosa et al., 2009; Etxeberria et al., 2015). It has also been reported to increase hepatic bile acid neosynthesis via gut microbiota remodeling, associated to the reduction of trimethylamine-N-oxide (TMAO) levels and atherosclerosis (Chen et al., 2016).

Other interesting polyphenols with prebiotic properties are anthocyanins. Positive effects against obesity and related alterations have been attributed to anthocyanin intake (from blueberries) (Meydani and Hasan, 2010). Anthocyanins are a major type of polyphenols, and have prebiotic properties since their low bioavailability make them likely to be metabolized by gut microbiota, and anthocyanins and their metabolites may exert a positive modulation on the intestinal bacterial population (Hidalgo et al., 2012). Relevant to this review is the described association between anthocyanins and BAT activation. In this sense, animal studies on supplementation with anthocyanins, or anthocyanin metabolites, have shown a relationship with thermogenic capacity of BAT and browning of WAT. More specifically, supplementation with cyanidin-3-glucoside (C3G) of genetically obese mice (C57BLKS/J-Leprdb/Leprdb) for 16 weeks resulted in reduction of weight gain, ameliorated glucose homeostasis impairment and hepatic steatosis, improved tolerance to cold exposure, and stimulated BAT activity and browning in subcutaneous WAT (You et al., 2017). Similarly, wild type C57BL/6J mice supplemented with C3G for 15 weeks were resistant to HF/high fructose diet induced obesity, associated with increased thermogenic capacity of BAT and browning of inguinal WAT, by increasing mitochondrial biogenesis and function (You et al., 2018). Altogether, these data reveal that C3G intake may be considered as a novel approach to prevent and treat obesity.

Other polyphenols are also gaining significance in relation to their anti-obesity effects due to their thermogenic effect. For instance, supplementation with dietary green tea extract (GTE) from leaves for 8 weeks to mice fed a HF diet significantly reduces HF-induced adiposity in both WAT and BAT, by reducing both adipocyte size in WAT and lipid droplet size in BAT. It is noteworthy that markers of browning were induced in WAT in GTE treated mice, whereas markers of whitening were reduced in the BAT of these animals, suggesting a role of browning and thermogenesis induction in the anti-obesity beneficial effects of GTE intake (Neyrinck et al., 2017). Another extract rich in polyphenols used in research is camu camu (*Myrciaria dubia*) (an Amazonian fruit) crude extract (CC). Daily treatment with CC in mice fed a HF diet for 8 weeks markedly reshapes gut microbiota and confers protection against obesity development by increasing energy expenditure, accompanied by *Ucp1* induction in BAT and WAT, together with other markers of browning (Anhê et al., 2018). Moreover, CC-treated mice showed altered levels and composition of circulating bile acids and changes in bile acid composition (with higher unconjugated secondary bile acids) (Anhê et al., 2018). Finally, transplantation of microbiota from CC treated mice to germ-free mice partially reproduced the beneficial effects of CC supplementation (Anhê et al., 2018). One more example is capsaicin, which has also shown prebiotic properties (Kang et al., 2017). Capsaicin supplementation to male Wistar rats results in the appearance of multilocular adipocytes positive for UCP1 and CIDEA in retroperitoneal WAT, and increased expression of browning markers in inguinal WAT (Mosqueda-Solis et al., 2018).

### Prebiotics of Carbohydrate Nature and Thermogenesis

Different types of carbohydrates are classified as prebiotics, and we focus on different examples with experimental data supporting their role in the regulation of adaptive thermogenesis.

In the group of non-digestible complex carbohydrates with prebiotic properties, pectins are an interesting example and, concretely, high-esterified pectin (HEP). Regarding structure, pectin is a complex heteropolysaccharide, made up of a series of covalently linked polymers, and the degree of methylation determines its usage by bacteria (Hamaker and Tuncil, 2014; Fak et al., 2015), with HEP importantly being fermented by gut bacteria. HEP is a main component of soluble dietary fiber in vegetables and fruits, and is recognized as a prebiotic (Lattimer and Haub, 2010; Hamaker and Tuncil, 2014), whose intake is associated with health-promoting effects on body weight, lipid metabolism, glucose homeostasis, etc. (Sanchez et al., 2008; Adam et al., 2015; Palou et al., 2015). Concerning the effect of HEP supplementation on BAT thermogenesis and WAT browning, we have developed studies in rats showing that chronic HEP supplementation (from apples) decreases energy efficiency and protects from fat accumulation and metabolic disturbances caused by maternal malnutrition, in part associated to increased thermogenic capacity in BAT and WAT, including the induction of UCP1 (unpublished manuscript in preparation). Therefore, HEP is a cheap, easy-to-get prebiotic with important effects on metabolic health, influencing thermogenic capacity. There are more examples of fermentable polysaccharides that have been related to adaptive thermogenesis modulation. Weitkunat et al. investigated how inulin and guar gum supplementation for 30 weeks affects the metabolic syndrome-related disorders associated to HF diet feeding in mice. The results showed that both inulin and guar gum affect gut microbiota composition but only inulin, and not guar gum supplementation, attenuates the HF diet induced body weight and fat gain, and significantly induces brown/brite markers in subcutaneous WAT (Weitkunat et al., 2017). In the same work, the authors also investigated the effect of SCFA, as will be discussed below (see section on postbiotics).

Another example of non-digestible, but in this case not complex, carbohydrate with beneficial physiological effects on metabolic health is epilactose (Murakami et al., 2015). Epilactose is a rare disaccharide proposed to prevent diet-induced obesity from studies in mice. Murakami et al. showed how HF fed mice supplemented with epilactose were protected against body and fat gain, and revealed greater *Ucp1* gene expression in BAT and, surprisingly, also in muscle; they also reported decreased gene expression of macrophage infiltration markers in epididymal WAT. Moreover, supplemented mice showed a different caecum SCFA profile, with increased acetate and propionate levels (Murakami et al., 2015).

Given the above evidence, it seems clear that different types of prebiotics can affect adaptive thermogenic capacity, although this is a relative by new field where more studies are needed. Furthermore, the underlying mechanisms explaining the relationship between prebiotic supplementation and the induction of thermogenesis and browning is still not fully clear, although some works are starting to focus on unraveling such mechanisms. Although not the only one, an important point of connection between thermogenic capacity and prebiotics, as we have already suggested here, is the production of secondary metabolites by resident bacteria after fermentation of prebiotics. For instance, as already pointed out, SCFA profile and secondary bile acids might be important metabolites derived from bacteria metabolism able to connect prebiotic supplementation to thermogenic capacity regulation in BAT and WAT. Although the importance of postbiotics has been relatively overlooked, scientific evidence of their beneficial health effects by BAT thermogenic activation and WAT browning is progressively increasing, as will be discussed in the next section.

## Impact of Postbiotics on BAT Thermogenesis and WAT Browning

Postbiotics (also known as metabiotics, biogenics, or metabolite/cell-free supernatants) are “soluble factors (products or metabolic byproducts) secreted by live bacteria or released after bacterial lysis” and they provide physiological benefits to the host (Aguilar-Toalá et al., 2018). There are multiple types of postbiotics with varied structures, such as SCFA, peptides, enzymes, teichoic acids, exo- and endo-polysaccharides, vitamins, etc. (Aguilar-Toalá et al., 2018). As key examples, here we discuss the scientific evidence of the link between BAT activation and WAT browning with bile acids and SCFA.

### Bile Acids and BAT Thermogenesis

Bile acids (BA) are synthesized in liver from cholesterol, as primary bile acids, which are cholic acid (3α,7α,12α-trihydroxy-5β-cholanoic acid, CA) and chenodeoxycholic acid (3α,7α-dihydroxy-5β-cholanoic acid, CDCA) in humans, while in rodents CDCA is metabolized to muricholic acids (less toxic) (Martinot et al., 2017). The synthesized primary BA are conjugated with the aminoacids glycine or taurine (forming bile salts), prior to their secretion, which is fundamental to their role of emulsification of lipids during digestion (Martinot et al., 2017). There are also secondary BA, which are produced by the metabolization of primary BA by gut microbiota; the most common secondary BA species are deoxycholic acid (DCA) and lithocholic acid (LCA), coming from CA and CDCA, respectively, although dozens of secondary BA are produced by the gut flora, increasing the diversity of possible secondary BA (Martinot et al., 2017). It should be taken into account that the profile of secondary bile acids might be modulated by environmental conditions which can affect gut microbiota composition. In this sense, in the work already commented above by Zietak et al. (2016), it is reported that cold exposure and diet are able to modify BA metabolism. For instance, cold acclimation at 12°C of C57BL6/J mice, after only 1 day, increases conjugated BA levels, related to reduced abundance of *Lactobacillus*, which possesses greater bile acid deconjugation capacity than other species (Zietak et al., 2016). Moreover, in this work, cold acclimation increased both the production of BA species and the expression of genes related to BA synthesis (Zietak et al., 2016).

BA regulate numerous metabolic pathways in the host via the nuclear receptor farnesoid X receptor (FXR) and the G-coupled receptor TGR5 (Martinot et al., 2017). Among these metabolic pathways, the regulation of thermogenesis is included. Watanabe et al. showed that TGR5 activation by BA promotes intracellular thyroid hormone activity and increases thermogenesis in BAT (Watanabe et al., 2006). Diet supplementation with CA to HF diet fed C57BL/6J mice prevents, and even reverts, diet induced obesity, related to increased energy expenditure and the induction of TGR5 receptor, which increases cAMP production, involved in increased expression of genes coding for proteins related to BAT activation (PGC1α and β, UCP1 and 3, 2 iodothyronine deiodinase—D2–, straight-chain acyl-CoA oxidase 1—ACO—and muscle-type carnitine palmitoyltransferase 1—mCPT1), therefore showing activation of thermogenesis in BAT (Watanabe et al., 2006). In this work, apart from primary BA, the authors showed, *in vitro*, the capacity of secondary BA (DCA) and taurine conjugates (of CA and DCA) to induce cAMP production in cells (related to TGR5 activation) (Watanabe et al., 2006). Therefore, this work demonstrates the capacity of both primary and bacteria-metabolized BA to potentially induce thermogenic activity. In another related paper, it was suggested that UCP1 expression is required for weight-reducing action of bile acids (Zietak and Kozak, 2016). Other studies relate BA to BAT activation (even in humans) (e.g., Teodoro et al., 2014; Broeders et al., 2015) although focused on primary BA. Since we are focusing on postbiotics, another interesting study, which we have formerly discussed, is the work by Somm et al. with β-Klotho KO mice. Here, the authors show that β-Klotho KO mice have a high production of CA in liver, resulting in an important excess of DCA (secondary BA) production by microbiota, suggesting that resistance of these KO mice to diet-induced obesity is mainly caused by the high production of DCA and its signaling through the receptor TGR5 (Somm et al., 2017). It is important to note that DCA is suggested to be especially responsible of the activation of TGR5 to stimulate BAT thermogenesis (Somm et al., 2017), related to its greater potency (compared with other BA) to activate TGR5 *in vitro* (Maruyama et al., 2002) and that, in fact, TGR5 is mainly considered as a receptor for secondary BA (Kawamata et al., 2003).

### Role of SCFA in BAT Thermogenesis and WAT Browning

SCFA are important metabolites produced by gut microbiota by the fermentation of non-digestible carbohydrates. SCFAs are monocarboxylic acids of 2–6 carbons and the main SCFA for their abundance in plasma and caecum are (in order from more to less abundant) acetate, propionate, and butyrate, as measured in humans and rats (Topping and Clifton, 2001; Jakobsdottir et al., 2013; Schonfeld and Wojtczak, 2016). It should be taken into account that the microbiota profile conditions the relative proportions of individual SCFA produced; namely this, *Bacteroidetes* mainly produce acetate and propionate, and *Firmicutes* mainly butyrate (Pascale et al., 2018). However, the relationship may not be that simple and different phyla or genera are not exclusively associated to the production of a specific SCFA, e.g., a positive correlation between relative abundance of *Firmicutes* and plasma acetate levels in humans has been shown (Moreno-Navarrete et al., 2018).

SCFA affect a variety of biological processes in multiple organs and tissues and have been described to play a role in different aspects of energy metabolism and body weight control. For instance, diet supplementation for 4 weeks with salts of butyrate (5%), propionate (4.3%), or acetate (3.7%) has been described to protect against diet-induced obesity and insulin resistance in mice fed a HF diet (Lin et al., 2012). Butyrate and propionate, but not acetate, were able to stimulate gut hormones and reduce food intake (Lin et al., 2012). Propionate has also been described to inhibit lipogenesis via fatty acid synthase down-regulation in the liver, while acetate can act as a lipogenic substrate, therefore the acetate/propionate ratio seems important for *de novo* lipogenesis (Conterno et al., 2011). Moreover, De Vadder et al. showed that propionate and butyrate induce the expression of gluconeogenesis related genes and activate intestinal gluconeogenesis through different mechanisms, and this has metabolic benefits such as reduced body weight and adiposity, and improvement in glucose homeostasis, as studied in rodents (De Vadder et al., 2014). *In vitro* experiments revealed that butyrate can directly activate the expression of intestinal gluconeogenesis related genes (coding for glucose-6-phosphatase—G6PC—and the cytosolic form of phosphoenolpyruvate carbocykinase—PCK1), while propionate requires the gut-brain circuit involving the free fatty acid receptor 3 (FFAR3) in mice to induce intestinal gluconeogenesis (De Vadder et al., 2014). Regarding acetate, the literature points toward a complex role. As has already been commented, acetate can act as a substrate for lipogenesis, but it has also been described to activate AMPK *in vitro* and to reduce the expression of key genes for gluconeogenesis and lipogenesis in liver of KK-A(y) diabetic mice supplemented with acetic acid, associated to reduced hyperglycaemia (Sakakibara et al., 2006). Strikingly, in the literature, there are both studies suggesting that acetate treatment induces an increase in lipogenic and adipogenic capacity (Hong et al., 2005; Ge et al., 2008; Aberdein et al., 2014) or the promotion of obesity through a gut-brain-β-cell axis (Perry et al., 2016; Trent and Blaser, 2016), and studies that suggest beneficial and protection-against-obesity effects, such as the one commented above and some others (e.g., see Kondo et al., 2009; Aoki et al., 2017, and see below). Therefore, the role of acetate is somewhat controversial. Regarding BAT thermogenesis and WAT browning, recent literature points to a role of some SCFA, as discussed below.

Concerning acetate and thermogenesis, new findings agree with the potential capacity of acetate to increase the activity of BAT or WAT browning in different models. In this sense, Hu et al. (2016) studied BAT of mice treated with sodium acetate (150 mM) in drinking water for 6 weeks, reporting an induction of the expression of genes coding for PGC1α and UCP1 as compared to controls, accompanied by morphological changes, including mitochondrial biogenesis. Moreover, treatment with acetate of immortalized brown adipocyte cells (IM-BAT), obtained by primary cell culture of BAT from C57BL/6 mice, induced adipogenesis and mitochondriogenesis, together with up-regulation of gene expression of key thermogenic markers, such as adipocyte protein 2 (AP2), PGC1α, and UCP1, depending on the activation of the free fatty acid receptor G protein-coupled receptor 43 (GPCR43) (FFAR2) (Hu et al., 2016). Moreover, the work of Weitkunat et al. (2017) has already been commented in the section on prebiotics of carbohydrate nature and thermogenesis; herein, the authors suggest an induction of beige markers (browning) in WAT driven by acetate. Other studies also suggest an involvement of acetate in WAT browning (Sahuri-Arisoylu et al., 2016; Moreno-Navarrete et al., 2018). Intraperitoneal injection of nanoparticules of acetate increases gene expression of brown adipocyte markers (UCP1 and PR domain containing 16—PRDM16) in subcutaneous WAT of HF diet fed mice, which might be partly responsible for the reduced obesogenic phenotype of these treated animals (Sahuri-Arisoylu et al., 2016). However, no BAT recruitment due to acetate treatment was found in this work (Sahuri-Arisoylu et al., 2016). On the other hand, a recent study in obese humans has shown a positive significant association between brown markers PRDM16, UCP1 and thyroxine deiodinase 2—DIO2—in subcutaneous WAT and the *Firmicutes* relative abundance, which seems to contribute significantly to the variance of these markers in this adipose depot (Moreno-Navarrete et al., 2018). Moreover, *Firmicutes* relative abundance associates positively with circulating acetate levels (Moreno-Navarrete et al., 2018). Therefore, the authors conclude that microbiota composition influences WAT browning in these obese subjects, probably through plasma acetate (Moreno-Navarrete et al., 2018). There is also *in vitro* evidence linking acetate with browning. For instance, 3T3-L1 adipocytes have been described to increase the expression levels of several browning markers after acetate treatment (UCP1, PRDM16, DIO2, CIDEA, and transmembrane protein 26—TMEM26—, among others) (Hanatani et al., 2016). The authors also provide *in vivo* evidence in mice and conclude that acetate might exert its anti-obesity effects through the dissipation of energy excess, at least in part (Hanatani et al., 2016). We have also performed *in vitro* experiments with murine primary cultures of adipocytes treated with acetate, showing that it induces thermogenic capacity in brown adipocytes and in recruited beige/brite adipocytes form subcutaneous WAT (unpublished manuscript in preparation). Therefore, different studies suggest that acetate might have an anti-obesity effect partly related to the induction of WAT browning, together with the recruitment of BAT, thus increasing energy expenditure capacity.

There is less evidence regarding adaptive thermogenesis capacity and other SCFA. Nevertheless, there is some evidence for butyrate as a thermogenic molecule. For instance, Gao et al. showed, among other effects, that dietary butyrate treatment in HF diet fed mice for 12 weeks induces the expression of UCP1 and PGC1α in BAT and mitochondrial function and biogenesis in BAT and muscle, accompanied by an improvement in insulin sensitivity (Gao et al., 2009). Besides, Li and collaborators demonstrated in mice that chronic butyrate supplementation prevents diet-induced obesity, hypertriglyceridemia, hepatic steatosis, and hyperinsulinemia, although mainly attributed to reduction of food intake (Li et al., 2018). Nevertheless, the authors also showed an activation of BAT, associated to increased fatty acid utilization from plasma triglycerides (Li et al., 2018). Moreover, it must be highlighted that butyrate treatment in mice has also been shown to promote BAT thermogenic capacity by activating UCP1 protein expression (and PCG1α) (Gao et al., 2009). In the work of Li et al., no evidence of browning in subcutaneous fat was found, suggesting the butyrate is not involved in browning induction (Li et al., 2018). Regarding propionate, indirect evidence for its involvement on adaptive thermogenic capacity is given by the fact that it can directly induce SNS activity via GPR41 (FFAR3) at sympathetic ganglia, correlated with energy consumption (Kimura et al., 2011). Moreover, in this work, GPR41 ablation (*Gpr41*^−/−^ mice) was associated with lower *Ucp1* expression in BAT. The authors suggest an important role of SCFA-GPR41 interactions regulating SNS as a mechanism accounting for the effects of diet and pre/probiotics on body homeostasis (Kimura et al., 2011).

Overall, despite some controversial results, SCFA, particularly acetate, seem to be proper candidates to continue to be tested as inductors of thermogenesis in BAT and browning in WAT, in order to reduce the detrimental health effects associated to diet-induced obesity. In this sense, human studies are essential to establish a strong association between SCFA and their beneficial effects at different levels.

### Role of Other Postbiotics on BAT Thermogenesis and WAT Browning

Apart from the postbiotics shown here, there are other various possibilities. For instance, we commented above that anthocyanins may have positive beneficial effects as anti-obesity factors and as thermogenesis and browning inductors (You et al., 2015, 2017) yet, interestingly, the metabolites produced by the utilization of anthocyanins by gut microbiota may also develop a role in host thermogenesis induction, similar to other postbiotics. In this sense, vanillic acid has been recently described to exhibit thermogenesis induction capacity (Han et al., 2018). Vanillic acid is one of the metabolites produced through the metabolization of anthocyanins by intestinal microbes (Keppler and Humpf, 2005). It has been reported that supplementation with vainillic acid for 16 weeks protects mice from HF/high sucrose diet induced obesity, by activating thermogenesis in BAT and browning in WAT (Han et al., 2018). These results, again, point out the capacity of postbiotics as BAT activators, and browning agents, in the treatment or prevention of diet-induced obesity. Another interesting postbiotic is 10-oxo-12(*Z*)-octadecenoic acid (KetoA). KetoA is a metabolite of linoleic acid produced by lactic acid bacteria in the gut able to stimulate SNS through activation of the ion channel transient receptor potential vanilloid 1 (TRPV1) in the gastrointestinal tract, concomitantly enhancing energy expenditure, related to activation of BAT function and inguinal WAT browning (Kim et al., 2017).

## Summary and Perspectives

**Figure 1:**
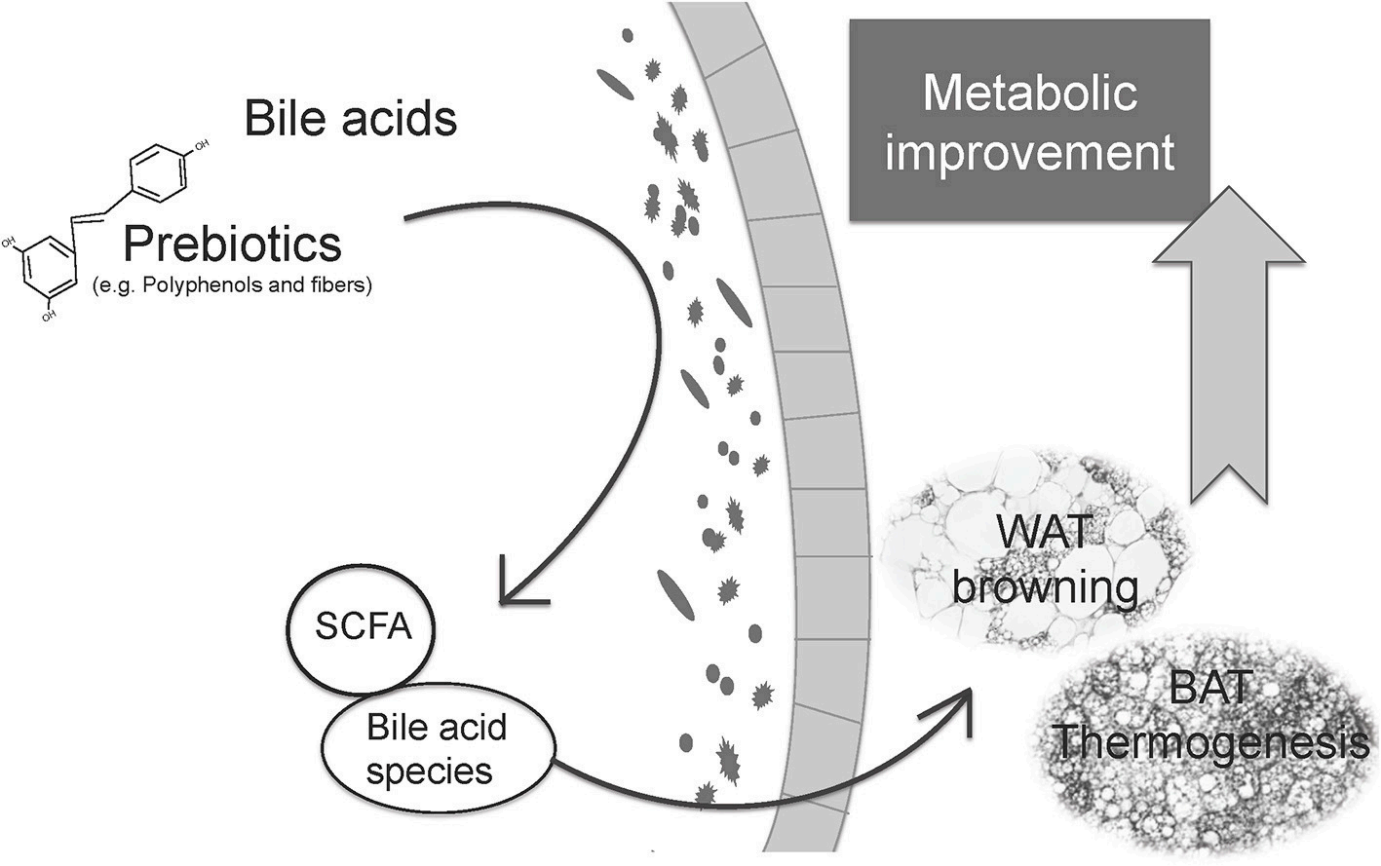
Relationship between prebiotics, microbiota modulation and postbiotics with thermogenic capacity regulation and the improvement of metabolic health. Prebiotics can modulate gut microbiota to promote eubiosis and the selective growth of beneficial species. Microbiota can metabolize prebiotics and primary bile acids to produce specific postbiotics, some of them with proven health benefits. The microbiota changes and the postbiotics produced can influence adaptive thermogenesis, promoting BAT recruitment, and WAT browning which, in turn, boost energy expenditure and contribute to the general improvement of metabolism, with associated health benefits.

The main bibliography and evidence of the different results shown in this review, supporting the role of prebiotics and postbiotics in adaptive thermogenesis regulation, is summarized in Tables 1, 2. Additionally, Figure 1 summarizes the central idea shown in this review, linking prebiotics, microbiota modulation, and postbiotics with BAT thermogenesis and WAT browning and the improvement of metabolism.

**Table 1:** Studies evaluated in this review of effects of prebiotics on adaptive thermogenesis by BAT recruitment and/or WAT browning.

**Type of Prebiotic**	**Prebiotic**	**Experimental design**	**Main results on BAT thermogenesis and WAT browning**	**References**
Phenolic compounds	Resveratrol	HF/high sucrose fed rats treated with resveratrol (15 mg/kg body/day), quercetin (30 mg/kg body/day) or both, for 6 weeks	Resveratrol and quercitin treatment: Appearance UCP1+multilocular cells in perirenal WAT ↑Expression of browning markers in perirenal WAT ↑Expression thermogenic markers *Cox-2* and UCP1 in BAT Resveratrol treatment: ↑Expression thermogenic markers *Cidea* and UCP1 in BAT	Arias et al., 2017
		HF/high sucrose fed Sprague-Daley rats treated with resveratrol (30 mg/kg body/day), for 6 weeks	↑Expression thermogenic markers in BAT, including *Ucp1*	Alberdi et al., 2013
		NMRI mice supplemented with resveratrol (2 mg/Kg body/day) during lactation (day 2–20 of life). HF diet from day 90 of life (for 10 weeks) vs. normal fat diet	↑Expression of browning markers in inguinal WAT in males ↑Multilocular UCP1+ adipocytes in inguinal WAT under normal fat diet ↓*Ucp1* expression in BAT	Serrano et al., 2018
	Anthocyanins	Obese male C57BLKS/J-Leprdb/Leprdb mice treated with Cyanidin 3-glucoside (1mg/ml) for 16 weeks	↑Energy expenditure ↑Expression of browning markers in sucbutaneous WAT ↑Expression of BAT thermogenic markers and BAT activity	You et al., 2017
		HF/high fructose fed male C57BL/6J mice treated with Cyanidin 3-glucoside (1mg/ml) for 15 weeks	↑Energy expenditure Better performance maintaining body temperature compared with died induced obese mice ↑Mitochondrial biogenesis and function ↑Expression of browning markers in inguinal WAT ↑Expression thermogenic markers in BAT	You et al., 2018
	Green Tea extracts	Male C57BL/6J mice fed with HF diet supplemented with green tea extract (0.5% green tea leaf extract) for 8 weeks	↑Expression of browning markers in subcutaneous WAT ↓Whitening in BAT ↑Appearance of UCP1+ adipocytes in subcutaneous WAT depot	Neyrinck et al., 2017
	Camu Camu (*Myrciaria dubia*)	Male C57BL/6J mice fed with HF/high sucrose diet supplemented with Camu Camu crude extract (200 mg/Kg) compared to Vitamin C (6.6 mg/Kg) for 8 weeks	↑Energy expenditure ↑Interscapular temperature (tendency) ↑Expression of browning markers in inguinal WAT ↑Expression thermogenic markers in BAT	Anhê et al., 2018
	Capsaicin	Male Wistar rats fed with HF/high sucrose and supplemented with Capsaicin (4 mg/kg body/day) for 8 weeks	↑Appearance of UCP1+ and CIDEA+ adipocytes in retroperitoneal WAT ↑Expression of browning markers in inguinal WAT	Mosqueda-Solis et al., 2018
Carbohydrates	Epilactose	Male C57/BL6 mice fed with HF diet, supplemented with Epilactore (10% by weight) for 8 weeks	↑UCP1 expression in BAT and muscle	Murakami et al., 2015
	Inulin and guar gum	Male C57BL/6JRj mice fed with HF diet supplemented with inulin or guar gum (7%) for 30 weeks	Inulin but not guar gum: ↑Body temperature ↑Expression of browning markers in subcutaneous WAT	Weitkunat et al., 2017

*The ability to induce or inhibit the assessed effects is indicated with ↑ or ↓ respectively. BAT, Brown adipose tissue; Cidea, Cell death-inducing DFFA-Like effector A; Cox-2, Cytochrome c oxidase subunit II; HF, High fat; UCP1, Uncoupling Protein 1; WAT, White adipose tissue*.

**Table 2:** Studies evaluated in this review of effects of postbiotics on adaptive thermogenesis by BAT recruitment and/or WAT browning.

**Type of postbiotic**	**Postbiotic**	**Experimental design**	**Main results on BAT thermogenesis and WAT browning**	**References**
Secondary BA	DCA	Male Klb^−/−^ mice fed with HF diet for 8 weeks	Effects associated to DCA: Thermogenesis and BAT stimulation	Somm et al., 2017
	DCA	Treatment with BAs to BAT cells *in vitro* from chow and HF fed C57BL/6J mice	Effects associated to DCA: ↑cAMP levels in BAT cells	Watanabe et al., 2006
SCFA	Acetate	Immortalized brown adipocytes cell line treated during differentiation with acetate (10 mM) or acute treatment 6 h (10 mM)	↑Expression of thermogenic markers both treatments (during differentiation and acute) ↑Mitochondriogenesis (during differentiation)	Hu et al., 2016
	Acetate	Male C57BL/6J mice supplemented with sodium acetate (150 mM) in drinking water for 6 weeks	↑Expression of thermogenic markers in BAT	Hu et al., 2016
	Acetate	Male C57BL/6JRj mice fed with HF diet supplemented with 5% of SCFA (10:1 Acetate/Propionate or 1:2.5 Acetate/Propionate) for 30 weeks	Effects attributed to acetate: ↑Body temperature ↑Expression browning markers in subcutaneous WAT	Weitkunat et al., 2017
	Acetate	Male C57BL/6 mice fed with HF diet treated with nanoparticle-delivered acetate (intraperitoneal injection three times per week) for 6 weeks	↑Expression browning markers in subcutaneous WAT No BAT recruitment	Sahuri-Arisoylu et al., 2016
	Acetate	34 morbidly obese humans (28 women and 6 men)	Relative abundance of *Firmicutes* is positively associated with browning markers in subcutaneous fat. Plasma acetate levels are positively associated with *Firmicutes* relative abundance, also linked to the brown marker PRDM16 mRNA in subcutaneous fat	Moreno-Navarrete et al., 2018
	Acetate	*In vitro*: 3T3L1 treated with sodium acetate (1 mM) during differentiation (day 0 to harvesting) *In vivo*: Obese diabetic male kk-Ay mice treated with acetate (0.6%) in drinking water for 16 weeks	*In vitro*: ↑Expression of browning markers *In vivo*: = Expression of thermogenic markers in BAT ↑Expression of browning markers in gonadal WAT = Expression of browning markers in subcutaneous WAT	Hanatani et al., 2016
	Butyrate	Male C57BL/6J mice fed with HF diet supplemented with sodium butyrate (5%) for 12 weeks	↑Cold tolerance ↑Energy expenditure ↑Expression of thermogenic markers in BAT ↑Mitochondrial function and biogenesis in BAT	Gao et al., 2009
	Butyrate	Male APOE*3-Leiden. CETP mice fed with HF diet supplemented with sodium butyrate (5%) for 9 weeks	↑Fat oxidation rate during day ↑Expression of UCP1 in BAT in BAT = Expression of browning markers in subcutaneous and gonadal WAT	Li et al., 2018
Anthocyanin metabolites	Vanillic acid	Male C57BL/6J mice fed with HF/high sucrose diet supplemented with vanillic acid (0.5%) for 16 weeks	↑Cold tolerance ↑Expression of thermogenic markers in BAT ↑Expression of browning markers in inguinal WAT ↑Mitochondriogenesis in BAT and WAT	Han et al., 2018
Linoleic acid metabolites	KetoA	Male C57BL/6 and TRPV1-deficient C57BL/6 mice fed with HF diet supplemented with KetoA (0.1%) for 10 weeks	↑*Ucp1* gene and protein expression in BAT ↑*Ucp1* gene and protein expression in inguinal WAT ↑Expression of thermogenic markers in BAT ↑Expression of browning markers in inguinal WAT These results depends on TRPV1 SNS dependent activation	Kim et al., 2017

*The ability to induce the assessed effects is indicated with ↑ while = indicates no significant changes. BA, Bile acids; BAT, Brown adipose tissue; DCA, Deoxycholic acid; HF, High fat; KetoA, 10-oxo-12(Z)-octadecenoic acid; PRDM16, PR domain containing 16; SCFA, Short chain fatty acids; SNS, sympathetic nervous system; TRPV1, Transient receptor potential vanilloid 1; UCP1, Uncoupling Protein 1; WAT, White adipose tissue*.

The incipient growth of studies suggesting a connection between microbiota and energy expenditure brings to light the potential of establishing new strategies against the pandemic of obesity. It is important to highlight the opportunity of the present review, since in the last 2–3 years numerous articles have been published studying the role of microbiota and the effects of prebiotics and postbiotics on the development of obesity and, more specifically, relating them to the activation of thermogenesis in BAT and browning in WAT. Microbiota suffers adaptations to the conditions of the host and responds to feeding, in such a way that it can be modulated by prebiotics conferring health benefits, which include the increase in thermogenic capacity, in both BAT and WAT (especially subcutaneous WAT). Therefore, boosting thermogenic capacity, and therefore energy expenditure, can be added to the list of the described beneficial health effects of prebiotics. Moreover, the products of microbiota metabolism, so-called postbiotics, are involved in the mechanisms that connect prebiotics and microbiota modulation with thermogenesis and protection against obesity and its associated metabolic disorders. Interestingly, the direct use of postbiotics, through dietary supplementation, can also be considered as an interesting strategy to combat obesity and improve host metabolism.

In conclusion, the use of both prebiotics and postbiotics, in order to induce adaptive thermogenesis in the treatment or prevention of obesity and metabolic disorders emerges as an interesting strategy which deserves further research. Taking into account the fact that an important part of this research has been carried out in animal models, more studies are needed in humans. Moreover, the use of prebiotics represents a physiological, and even easy and affordable, way to improve health and energy metabolism. Overall, it is worthwhile to continue investigating this connection, given its therapeutic potential.

## Author Contributions

All authors designed the review. BR, MP, and AMR wrote the manuscript. AP and AMR revised the definitive version. All authors read and approved the final manuscript.

### Conflict of Interest Statement

The authors declare that the research was conducted in the absence of any commercial or financial relationships that could be construed as a potential conflict of interest.
